# *SegWay*: A simple framework for unsupervised sleep segmentation in experimental EEG recordings

**DOI:** 10.1016/j.mex.2016.02.003

**Published:** 2016-02-21

**Authors:** Farid Yaghouby, Sridhar Sunderam

**Affiliations:** Department of Biomedical Engineering, University of Kentucky, Lexington, KY, USA

**Keywords:** Sleep scoring, EEG, EMG, Clustering, Hidden Markov model, Bout duration, Vigilance state, Sleep–wake, Behavior, Classifier

## Abstract

Sleep analysis in animal models typically involves recording an electroencephalogram (EEG) and electromyogram (EMG) and scoring vigilance state in brief epochs of data as Wake, REM (rapid eye movement sleep) or NREM (non-REM) either manually or using a computer algorithm. Computerized methods usually estimate features from each epoch like the spectral power associated with distinctive cortical rhythms and dissect the feature space into regions associated with different states by applying thresholds, or by using supervised/unsupervised statistical classifiers; but there are some factors to consider when using them:

• Most classifiers require scored sample data, elaborate heuristics or computational steps not easily reproduced by the average sleep researcher, who is the targeted end user.
• Even when prediction is reasonably accurate, small errors can lead to large discrepancies in estimates of important sleep metrics such as the number of bouts or their duration.
• As we show here, besides partitioning the feature space by vigilance state, modeling transitions between the states can give more accurate scores and metrics.

An unsupervised sleep segmentation framework, “*SegWay*”, is demonstrated by applying the algorithm step-by-step to unlabeled EEG recordings in mice. The accuracy of sleep scoring and estimation of sleep metrics is validated against manual scores.

## Methods

### Approach

In our simple framework for unsupervised sleep segmentation, dubbed “*SegWay*”, unlabeled data are first separated into clusters that correspond to each of the vigilance states based on their location in the feature space. This clustering is seen to give reasonably accurate predictions of vigilance state from sample feature measurements. But correctly predicting scores for a large proportion of epochs of data alone does not guarantee that state transitions are accurately identified. Therefore, in addition to clustering the data to determine the vigilance states, dynamical transitions between the states are modeled using a Markov chain. The two aspects in combination—i.e., observations conditioned on latent states and probabilistic state transitions—is known as a hidden Markov model [1]. Sleep scores are predicted from the HMM, which can be used to infer the sequence of vigilance states underlying a time series of sample points in the feature space. The use of the HMM leads to improved prediction of state and estimation of dynamical attributes like the proportion, number of bouts, and mean bout duration of each vigilance state, which are of interest in sleep research [2]. The HMM, when fitted to baseline data, can then be used to score future recordings in the same animal under other conditions. Analysis steps within this framework are demonstrated on the Matlab environment with sample data and visual aids where appropriate. The associated script and sample data are as supplementary material with this article.

### Step 1. Animal procedures, EEG/EMG measurements, and manual sleep scoring

EEG and EMG measurements were recorded for 24 h each (7 a.m.–9 p.m. Light, 9 p.m.–7 a.m. Dark) in C57BL/6J mice (*n* = 18, 8–10 weeks old, 24–29 g weight) from Jackson Labs (Bar Harbor, Maine) using procedures approved by the Institutional Animal Care and Use Committee (IACUC) of the University of Kentucky. Each animal was stereotaxically implanted under isoflurane anesthesia with a headmount (Pinnacle Tech., Lawrence, Kansas) that was affixed to the skull by four screws, of which two served as epidural EEG electrodes (one frontal and one parietal) and the other two as common reference and ground respectively. The frontal screws were located 3.0 mm anterior to bregma. Two silver wires extending from the rear of the headmount were inserted into the nuchal muscle to record bipolar EMG. The reader is referred to previous work [3] for further details of the implantation technique, which is standard practice for such behavioral recordings in rodents.

After allowing the animal to recover for about two weeks, it was placed for monitoring in a 7″ × 7″ plexiglass cage with bedding and free access to food and water. A 100× preamplifier was clipped to the headmount and conveyed the EEG and EMG signals via a slip-ring commutator to a biosignal amplifier (Pinnacle), which further amplified (50×), bandpass-filtered (0.5–100 Hz for EEG, 10–100 Hz for EMG) and sampled the data at 400 Hz and 16-bit resolution for storage and analysis on a computer. Time-stamped digital video of the animal was recorded using a webcam with infrared illumination to assist in scoring of behavior in both Light and Dark conditions.

A human rater scored each 24-h recording in 4-s epochs as Wake, NREM, and REM vigilance states using standard criteria. Sleep and Wake were differentiated based on muscle tone measured by the EMG. During sleep, when the EMG is low in amplitude, epochs were labeled as NREM and REM depending on whether EEG spectral power was concentrated in the delta (0.5–4 Hz) or theta (6–9 Hz) band respectively and quasirhythmic oscillations in these frequency ranges were observed. The human rater's scores were stored for later use in the validation of the *SegWay* algorithm. The EEG/EMG recordings and manual scores were split into Light (7 a.m. to 9 p.m.) and Dark (9 p.m. to 7 a.m.) data sets so that the Light data could be used for developing the classifier and the Dark data for validation as out-of-sample data.

### Step 2. Selection and extraction of EEG/EMG signal features

All analysis from this step onward was performed on Matlab™ (Mathworks, Natick, Massachusetts). Three features were extracted in each recording: 1. The r.m.s. power of the EMG after bandpass-filtering from 80 to 100 Hz, which expresses muscle tone and differentiates sleep from wakefulness; 2. The ratio of the mean-squared signal power of the frontal EEG after bandpass-filtering in the delta (0.5–4 Hz) and theta (6–9 Hz) bands, respectively, which helps differentiate REM from NREM; and 3. The ratio of mean-squared frontal EEG power in high frequency (9–45 Hz) and low frequency (0.5–9 Hz) bands, respectively, which further distinguishes REM. A 3rd order Butterworth filter was used in each case. These signal features were estimated in each 4-s epoch of the recording and used as inputs for classification of the three vigilance states. As a preprocessing step, each feature time series was smoothed using a four-point (16 s) moving average and scaled logarithmically to reduce noise and skewness in the distribution, which is a common feature of signal power estimates.

***Comments:*** Various other signal feature sets that separate Wake, NREM, and REM are described in the literature on sleep scoring in rodents (reviewed in [4]), but an EMG power estimate is frequently used to differentiate Wake from sleep, and one or more EEG features to separate REM from NREM on the basis of delta, theta, alpha, and other commonly observed cortical rhythms. These features can be substituted for the ones presented here, which we have found useful, and are expected to work in a more or less similar manner with little or no modification required.

### Step 3. Clustering of unlabeled signal features into vigilance states

**Fig. 1:**
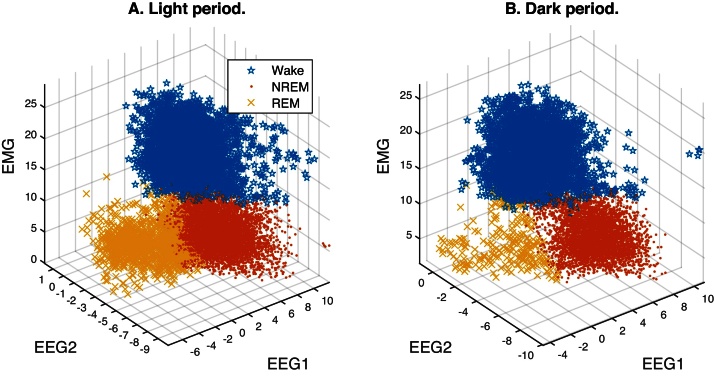
Clustering of EEG/EMG feature samples into vigilance states. (A) Signal features (EEG1 and EEG2 are delta/theta and hi/lo power ratios) extracted from 4-s epochs of a 14-h recording in a mouse during the Light period were clustered into three components using the *k*-means algorithm. The clusters are distinctly separated in the feature space and showed a 91.6% agreement with manually scored vigilance state (Wake, NREM, REM). (B) Each feature sample extracted from a 10-h Dark period was mapped to the cluster from (A) with the nearest centroid. Agreement between vigilance state predicted in this manner and manual scores was 93.5%.

Fig. 1 shows sample feature data extracted from one animal's EEG/EMG recording. The samples are scattered over the feature space, but distinct clouds or clusters of points are visible that are found to correspond to each vigilance state. Here we use the *k*-means algorithm [5] to segregate feature samples in the Light period into three clusters. This is done in two stages as described below: the first to segregate sleep from wakefulness, and the second to partition REM and NREM within sleep. The algorithm's goal is to identify prototypes or *centroids* for each of several clusters so as to minimize the ratio of the within-cluster distance of each sample from its cluster centroid relative to the mean distance between all the centroids: i.e., a variance criterion (VC). It starts with the user specifying the number of desired clusters. Seeds are then selected for the centroid of each cluster (by a *k*-means++ algorithm) and its members determined from their distance relative to all the centroids. The VC is computed and the centroids updated based on their members in the current iteration. When the VC converges to an optimum and does not improve with further iteration, the algorithm returns the final cluster centroids and their members.

In order to increase the likelihood of a favorable partition (i.e., one that best separates samples by vigilance state) we first use the *k*-means algorithm to partition the r.m.s. EMG feature values into two clusters, sleep and Wake, and then apply it again to separate the sleep cluster into two sub-clusters corresponding to REM and NREM based on the delta-theta power ratio feature alone. The clusters in Fig. 1 are colored by their final membership after application of the *k*-means algorithm.

We find that this simple unsupervised clustering procedure labels EEG/EMG features in a way that agrees substantially with manual scores. It should be noted that even expert human raters can disagree by 5–10% or more [6], [7], so this limits the performance to be expected when comparing the algorithm with any one rater. Perfect agreement with manual scoring is neither feasible nor desirable, since the model may be overfitting the data. For the sample animal's Light data (Fig. 1A), overall agreement was 91.6% with >90% high sensitivity and specificity for each state. The *k*-means model built from the Light period data was then used to classify data from the Dark period by assigning labels to each epoch based on which of the three centroids is nearest to it in the feature space and found to perform just as well (Fig. 1B): overall accuracy was 93.5%; sensitivity and specificity for Wake and NREM were similarly high but REM sensitivity was low (60%) and specificity was moderate (85%).

***Comments:*** Many unsupervised algorithms, for instance Gaussian mixture models and linkage trees, are available for clustering unlabeled sample data, and may work just as well as the *k*-means. The clustering approach presented above offers a quick way to predict vigilance state in unlabeled data. The only assumption made is that all three states are reasonably well-represented in the sample data to be modeled (but not necessarily in future samples) since this affects the accuracy of the estimated centroid locations. The proportion of each vigilance state in the sample was assayed fairly well: 34, 55, and 10% versus 37, 55, and 8% from manual scores for Wake, NREM, and REM respectively in the Light period (similar high accuracy in the Dark period).

**Fig. 2:**
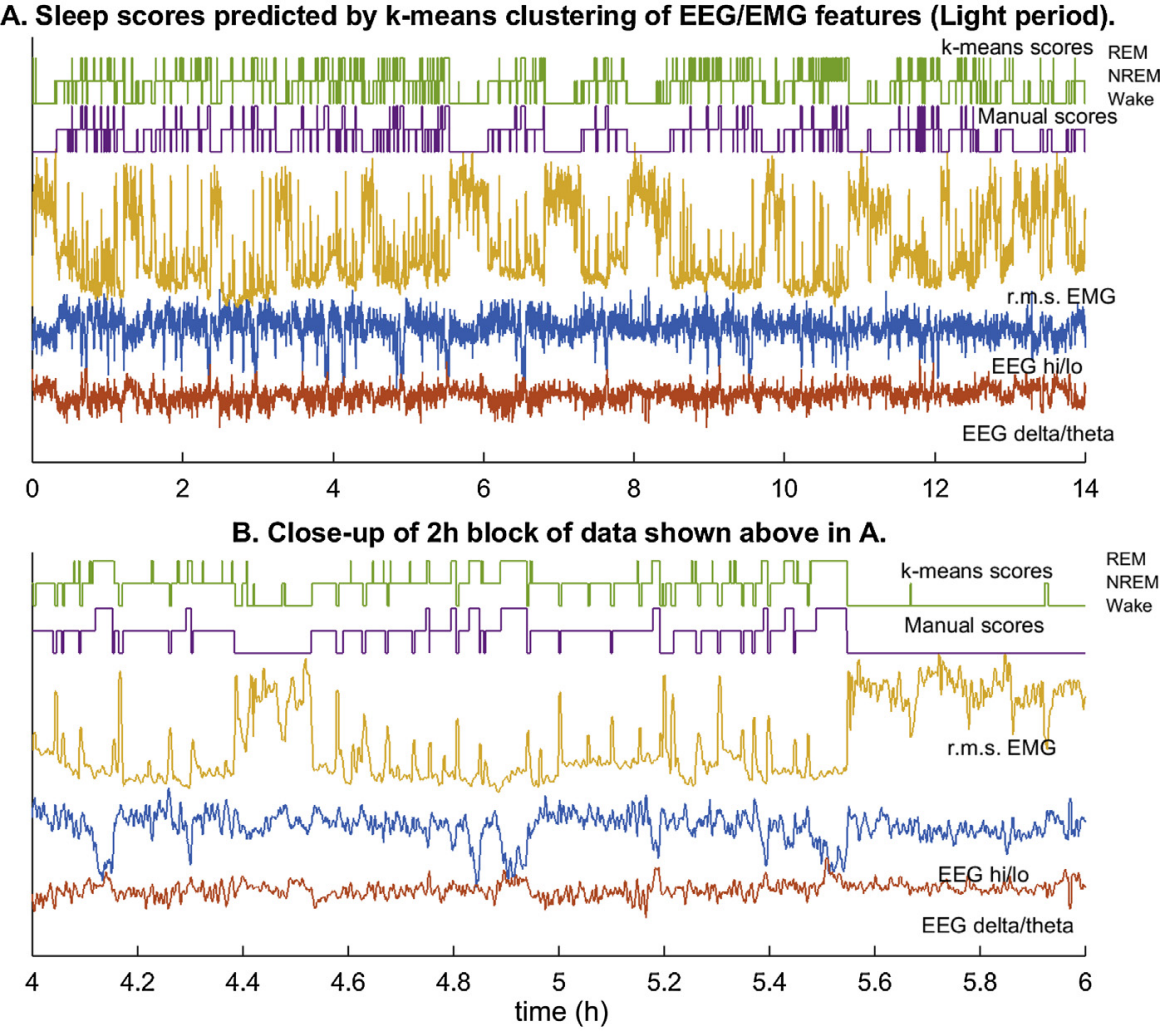
Sleep scoring by the *k*-means algorithm. (A) The time series of features estimated from 4-s samples of data in the Light period for a mouse, the r.m.s. EMG power (saffron), EEG delta/theta power ratio (red), and EEG hi/lo power ratio (blue), are displayed along with manual (violet) and *k*-means-predicted scores (green) of the sequence of vigilance states. The three levels of each hypnogram correspond to Wake, NREM, and REM. (B) A 2-h segment of data from (A) expanded to show detail. Multiple brief arousals and REM transitions during sleep and occasional brief episodes of NREM during wakefulness are marked by the algorithm that are not present in the manual scores. These errors mainly affect estimates of bout number and duration.

But a closer look at the hypnograms—i.e., the sequences of epoch state labels (manual and predicted) for the recording—reveals a serious limitation (Fig. 2). Even as the predictions appear to track the most likely state (per manual scoring) fairly closely over time, there are frequent errors—particularly arousal and REM transitions that are not recognized in the human scores—that could be attributed to noise or variability in the feature values within a state. These errors may be for a small fraction of all epochs, but they may cause the classifier to miss important state transitions or introduce false transitions where there are none, thus introducing large errors in estimates of bout number or duration.

The number of bouts or their durations for each vigilance state are important metrics that characterize sleep dynamics in experimental investigations (e.g., [2]) but sleep scoring methods do not report how accurately they are estimated by computer algorithms [7], [8], [9], [10] with some exceptions [11]. Here we find that *k*-means clustering of the vigilance states injects brief episodes of NREM into prolonged Wake bouts and spurious episodes of REM or brief arousal during sleep (Fig. 2). We speculate that such errors will occur with most published algorithms even if the reported accuracy is significantly greater than 90%.

### Step 4. Modeling vigilance dynamics from signal features using HMMs

In order to improve the estimation of bout number and duration, it is necessary to model the dynamics of vigilance state across transitions in a way that allows state scores to be predicted for *sequences* of observed EEG/EMG features rather than individual samples. A hidden Markov model (HMM) is capable of doing just this [1].

The HMM is based on the concept of a Markov chain, i.e., a graphical model in which each node is a discrete state that can make transitions—identified by the edges—at random times to other states with probabilities that depend only on the current state (known as the Markov property). When the states of the Markov chain correspond to the vigilance states, such a model would describe the dynamics of transitions between Wake, NREM, and REM in the form of a matrix of state transition probabilities. But since the state is not directly observable, it must be inferred from measurements such as the EEG/EMG signal features. An HMM specifies the distribution of feature values conditioned on each state of the Markov chain, or the *conditional*. Elementary rules of probability (Bayes theorem and the chain rule) are combined in the form of a simple but powerful technique, known as the Viterbi algorithm [1], to decode the sequence of HMM states—assuming the HMM's parameters (transition matrix and conditional distributions)—that are most likely to have generated a sequence of observed feature values.

First, we need to fit the HMM to the available feature data. To do this, we model the distribution of sample data in the feature space, again using the *k*-means algorithm, but this time with a larger number of clusters (between 3 and 15). The algorithm selects the partition with the least number of clusters *M* needed for a variance criterion (similar to the *F* statistic) to exceed 90%. This is one way to select a parsimonious number of centroids that adequately represent the scatter of data observed in the feature space, so that any sample point observed in the future is likely to be close enough to a centroid and belong to that cluster.

**Fig. 3:**
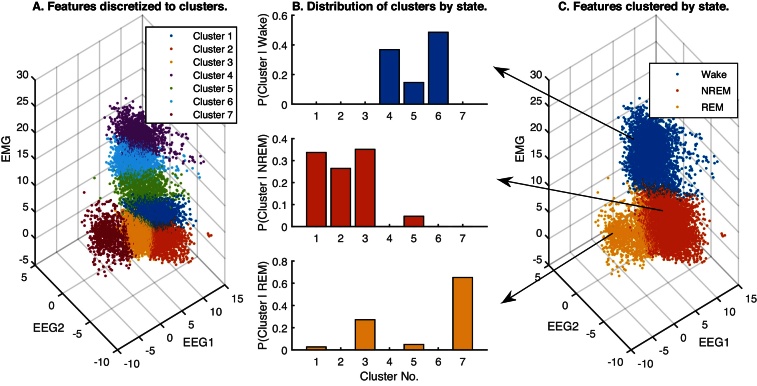
Components of the discretized observation HMM of vigilance dynamics. (A) Sample EEG/EMG features (EEG1 = EEG delta/theta power ratio; EEG2 = EEG hi/lo power ratio) extracted from the Light period are separated into seven observation clusters by the *k*-means algorithm. (B) The probability distribution of these clusters conditioned on each vigilance state (Wake, NREM, REM). (C) Vigilance state determined by a three-state *k*-means clustering of the same data as in (A). The HMM is specified by fixing the vigilance states, computing the probabilities of transitions between the states from sample data, and computing the conditional distributions of the discretized observations. The sequence of states underlying future observations can then be decoded by the HMM using the Viterbi algorithm.

The partitioning or mapping of feature values to clusters described above becomes a discretized observation space with a relatively small number, *M*, of centroids. Each cluster acts as a bin, akin to that used in a discrete observation HMM, except that any point in the feature space is now mapped to the cluster of the nearest centroid. The member samples of each cluster are then used to compute the discrete conditional probability distribution of the samples for each of the three vigilance states (Fig. 3). Then we take the original output of the *k*-means algorithm, which clusters the data into three vigilance states, and estimate the relative frequencies of transitions between the states to make up the 3 × 3 state transition matrix. The combination of conditional distribution and state transition matrix comprise the parameters of an HMM with discretized observations that captures the dynamics of vigilance state transitions. This initial guess of the parameters is refined by applying the Baum–Welch algorithm, which is an E–M procedure for performing maximum-likelihood estimation [1] that converges to a local optimum. In our code, the user has the option of applying the Baum–Welch optimization or using the initial guess HMM to compare the two. We have found that the two-stage *k*-means procedure for deriving the initial guess produces better results with the Baum–Welch algorithm than starting with multiple random initial guesses and choosing the solution with greatest log-likelihood. We demonstrated a similar approach for segmentation of human sleep recordings in a previous study [12].

***Comments:*** There are well-documented approaches in the literature for modeling observations using HMMs that either: 1. Assume a model for the conditional distribution—usually a multivariate Gaussian or mixture-of-Gaussians—in each state [13]; or 2. Separate the feature space into bins of equal volume, and model the conditional as a discrete probability distribution for each state [14]. While the first approach assumes a continuous distribution over the feature space and can assign a probability density to any point in it, a sparse sample of observations can lead to poor estimates of parameters (e.g., ill-defined covariance matrices) for the conditional density functions. The latter discrete approach has its own drawbacks: fine-grained bins may be needed to better estimate the conditional, but this leads to many empty bins for sparsely sampled data and a prohibitively large parameter space, whose size is proportional to the number of bins. Our implementation is a hybrid of both approaches: firstly, observations of features are discretized, but by mapping them to clusters based on a statistical criterion and not by binning the feature space; and secondly, the conditional of the HMM is a discrete probability distribution of the symbolic clusters to which observations are mapped rather than parametric density functions of the continuous-valued observations. These features make it a *discretized* observation HMM rather than a discrete or continuous observation HMM.

### Step 5. Decoding the state sequence from EEG/EMG feature time series

**Fig. 4:**
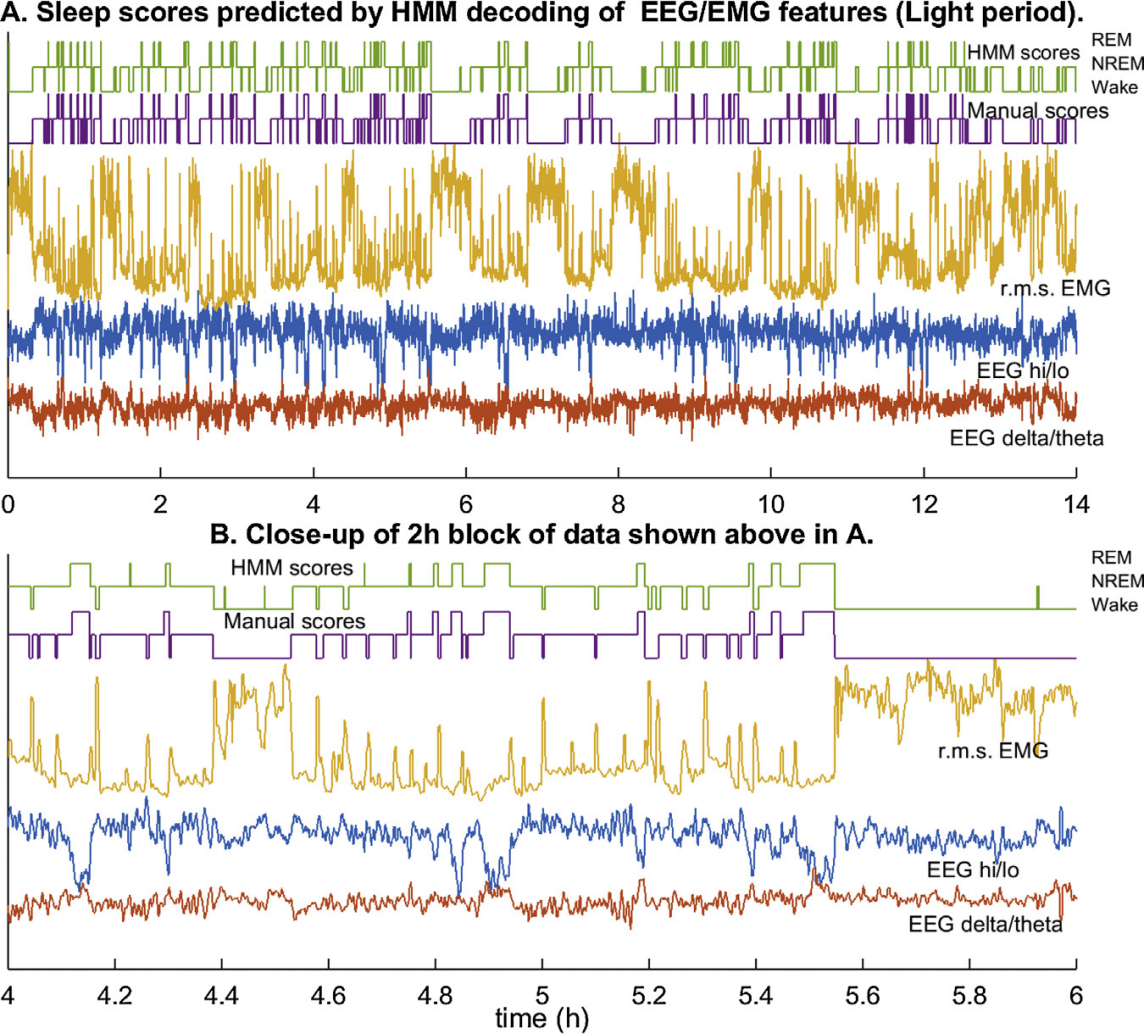
Sleep scoring by the discretized observation HMM (Light period). (A) The time series of signal features estimated from 4-s samples of data in the Light period for a mouse, the r.m.s. EMG power (saffron), EEG delta/theta power ratio (red), and EEG hi/lo power ratio (blue), are displayed along with the manual (violet) and HMM-predicted (green) sequence of vigilance states. The three levels of each hypnogram correspond to Wake, NREM, and REM. (B) A 2-h segment of data from (A) expanded to show detail. The HMM rarely generates predictions of arousal or REM that are not also present in the manual scores. This improves estimates of sleep metrics over values predicted from the *k*-means sleep scoring.

**Fig. 5:**
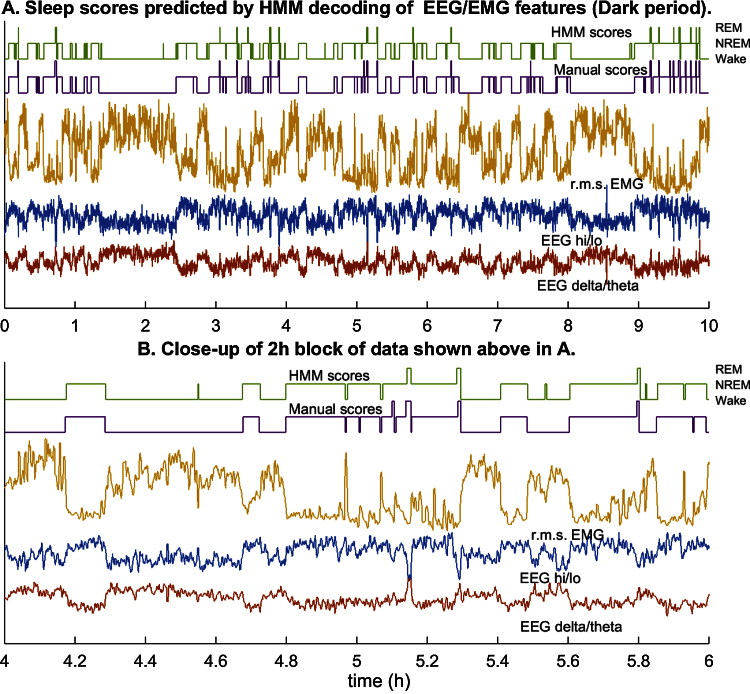
Sleep scoring in the Dark period by the discretized observation HMM fitted to data in the Light period. (A) The time series of signal features estimated from 4-s samples of data in the Light period for a mouse, the r.m.s. EMG power (saffron), EEG delta/theta power ratio (red), and EEG hi/lo power ratio (blue), are displayed along with the manual (violet) and HMM-predicted (green) sequence of vigilance states. The three levels of each hypnogram correspond to Wake, NREM, and REM. (B) A 2-h segment of data from (A) expanded to show detail.

After estimating the HMM, the popular Viterbi algorithm [1] is used to decode the state sequence from the time series of features. The Viterbi algorithm finds the state path, within the constraints imposed by the model parameters, that is most likely to have generated the entire sequence of observed features in the unlabeled data. This is essentially different from classification methods that only model differences between states in the feature space, which means that they optimize the separation based on the conditional distribution of features and not on dynamical changes in vigilance state. Application of the Viterbi algorithm to the Light data set shows that although the accuracy has barely increased (90%) and is actually lower than that of *k*-means for the Dark period (91% vs. 93%), prediction noise in the time sequence of states is visibly reduced (Fig. 4, Fig. 5). There are only rare instances of false NREM during prolonged Wake, and few false REM episodes or brief arousals during sleep.

***Comments:*** One limitation found here in using HMMs is that while they avoided generating false arousals in sleep, they also missed several genuine arousals marked by the human rater. This is attributed to the fact that both brief arousals in sleep and prolonged bouts of Wake are modeled as a single state when in reality they are dynamically distinct and operate on different timescales. In a future implementation, we hope to alleviate this behavior using a hierarchical HMM that first differentiates between prolonged sleep and wake bouts, and then models transitions between NREM, REM, and arousal within sleep. Another way to address this problem is to use a generalized Markov model framework, in which dwell time distributions for each state are explicitly modeled, and further assuming a form for the distribution flexible enough to accommodate (and test for) significant deviations from a geometric distribution [15].

### Validation of the SegWay algorithm on a cohort of mice

The SegWay algorithm that we have demonstrated in a sequence of steps using sample data from one animal was applied to 24-h recordings in a cohort of eighteen animals and the predicted sleep scores compared with manual scores. The results (Table 1), show that the initial *k*-means clustering of the feature space is a quick and easy way to obtain reasonably accurate predictions of the vigilance states of individual samples with about 90% overall agreement when Light data is clustered and the model is tested on Dark data. Agreement is comparable but slightly poorer when the “Dark model” is tested on Light data. Moreover, the output is noisy and has a tendency to make isolated false predictions—observable as “flicker” in the model output—in the middle of an ongoing state (Fig. 2). Note that the Light model gives better agreement (Table 1) when applied to the Dark data than the Dark model itself. This is attributable to the difference in behavior between these periods: in the Dark period, mice are relatively active and spend much less time in sleep and very little in REM (sometimes as low as 1%). This gives poor estimates of the distribution of REM for the unsupervised models. On the other hand, all three vigilance states are more evenly represented in the Light period, and their distributions are therefore better estimated. This leads to more accurate scoring even in the Dark period. This underlines the importance of good baseline data when using the model for future out-of-sample analyses.

**Fig. 6:**
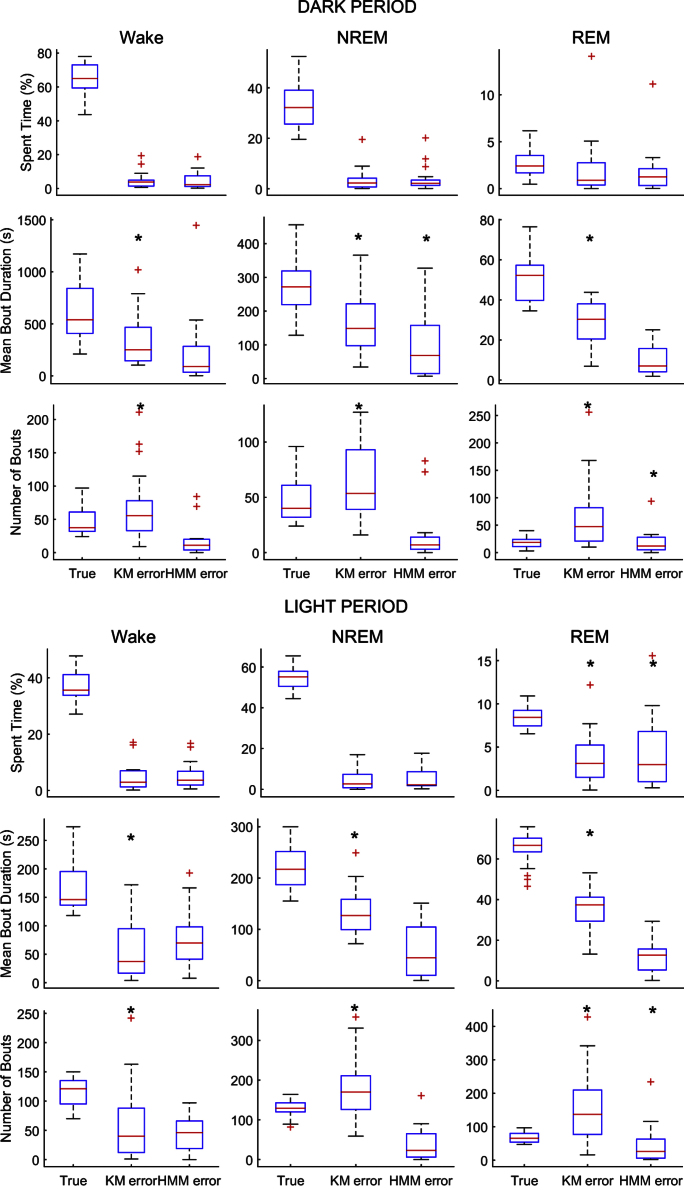
Distribution of errors in sleep metrics estimated by *k*-means (KM) and HMM models relative to manual estimates (True) in 18 mice. Models estimated from Light data were used to classify Light (lower) and Dark (upper) data for each animal. Asterisk (*) indicates that model-predicted values are significantly different from manual estimates (Wilcoxon signed-rank test; *p* < 0.01). This was the case for most *k*-means estimates but not so for most HMM-estimated values.

**Fig. 7:**
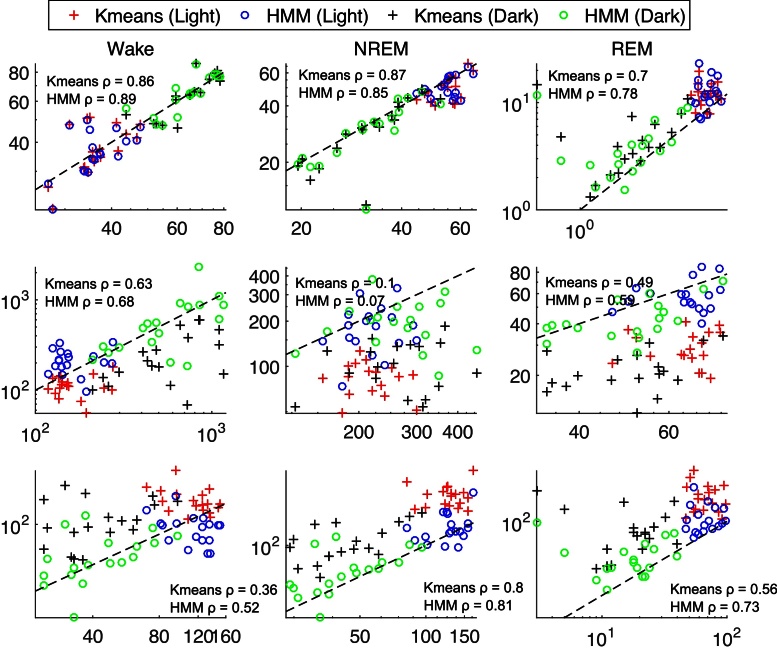
Estimates of sleep metrics (ordinate) from *k*-means and HMM sleep scores compared with estimates from manual scores (abscissa) in 18 mice. Models fitted to Light data were applied to both Light and Dark data. Abscissa and ordinate are equal along the dashed line. Spearman's rank correlation coefficient is specified for *k*-means and HMM in each panel (Light/Dark data pooled). HMM estimates of Spent Time (%) are strongly correlated with manual estimates; the correlation is relatively poor for Mean Bout Duration except in Wake; and Number of Bouts shows a moderate-to-strong correlation. In contrast, the *k*-means algorithm clearly underestimates mean bout duration and overestimates the number of bouts for all three states.

When the distribution of samples in the feature space is modeled using a larger number of discrete clusters, and these clusters are treated as observations generated by an HMM of the original three clusters corresponding to vigilance states (Fig. 3), predictions of sleep scores did not appear to be any more accurate. But the prediction noise is visibly reduced and the classifier is less likely to generate false positives (Fig. 4, Fig. 5). This is borne out by the cohort data in Fig. 6, Fig. 7, in which the *k*-means and HMM models estimated from Light data are applied separately to Light and then Dark data, and confirms that incorporating dynamics into sleep classifiers is essential for predicting metrics that characterize the dynamics of sleep.

## Conclusions and recommendations

1. The litmus test for a sleep scoring algorithm should be the accuracy with which sleep metrics are estimated from the predicted sequence of scores. Comparison of model-predicted and manual scores (Fig. 4, Fig. 5 and Table 1) and the corresponding estimates of sleep metrics (Fig. 6) suggests that the HMM built from observations discretized by *k*-means clustering gives much more accurate estimates of the number of bouts and mean bout duration of each vigilance state.
2. SegWay is intended as a tool for sleep analysis that, when given a baseline recording, will model the differences between vigilance states in an *unsupervised* manner—i.e., without the need for scored training samples—with reasonable accuracy. Performance is therefore dependent on the availability of a representative baseline recording in which all vigilance states and their transitions are reasonably well represented. For this purpose, we recommend the use of several hours of data from the Light period in rodents since they are nocturnal by nature. Another reason for attempting an unsupervised approach is to avoid incorporating a human rater's bias into the scoring.
3. Once fitted to a baseline EEG recording for an animal, SegWay can then be used to score data from the same animal under an experimental condition to evaluate effects on sleep characteristics. Here we have used data from the Dark period, in which mice are much more active, to test whether sleep can be scored out of sample by the algorithm. But it certainly does not show how it would work under sleep restriction or after treatment with a vigilance-modifying drug (e.g., benzodiazepine). Further study is indicated to validate the ability of the algorithm to score sleep under such conditions.
4. We have emphasized the use of SegWay for scoring baseline and experimental data separately for individual animals. It would of course be interesting to see how each animal's model generalizes to other animals. It is to be expected that performance will drop due to inter-subject variability and signal quality: for instance, placement of EMG electrodes can affect perceived muscle tone and the contrast between sleep and wakefulness. The consequent shift in EMG power, which is not an easy variable to normalize, can cause the locations of clusters in the feature space to differ from those for the training animal. In our analysis, we found that cross-validation of each animal's model on another animal's data was only about 70% accurate in the mean, clearly inadequate for most experimental purposes. We would therefore not recommend its use in this manner unless suitably normalized features can be found that do not significantly alter the distribution of the data.

## Supplementary material

A Matlab file segway_sleep.m, and sample Light and Dark feature data (segway_sample_data.mat), are available as supplementary material for readers who wish to use the methodology described here for their own purposes. The authors request that users cite this paper when using this material.

## Figures and Tables

**Table 1:** Accuracy of vigilance state prediction by *k*-means and HMM classifiers assessed against manual scores. All numbers reported in % as mean ± standard error (*n* = 18 mice).

	*k*-means			HMM		
	Sensitivity	Specificity	Overall agreement	Sensitivity	Specificity	Overall agreement
Light period (train)						
Wake	89 ± 1	92 ± 2		89 ± 1	93 ± 2	
NREM	86 ± 2	93 ± 0.8	87 ± 1	87 ± 2	93 ± 0.6	88 ± 1
REM	89 ± 2	95 ± 0.7		89 ± 1	95 ± 0.9	
Dark period (test)						
Wake	94 ± 1	90 ± 3		95 ± 1	90 ± 3	
NREM	87 ± 3	97 ± 0.5	92 ± 1	87 ± 3	97 ± 0.5	92 ± 1
REM	84 ± 3	97 ± 0.7		81 ± 2	98 ± 0.6	

Dark period (train)						
Wake	91 ± 2	96 ± 0.9		89 ± 2	97 ± 0.9	
NREM	87 ± 1	97 ± 0.6	90 ± 1	90 ± 1	97 ± 0.6	90 ± 1
REM	94 ± 1	93 ± 1		88 ± 2	92 ± 1	
Light period (test)						
Wake	75 ± 3	98 ± 0.6		76 ± 3	98 ± 0.7	
NREM	83 ± 2	92 ± 0.6	81 ± 2	87 ± 2	93 ± 0.9	83 ± 1
REM	95 ± 0.7	85 ± 2		88 ± 2	87 ± 2	
